# EHMTI-0019. Exploratory research of headache from psychiatric opd patients via biofeedback

**DOI:** 10.1186/1129-2377-15-S1-E40

**Published:** 2014-09-18

**Authors:** S Wang

**Affiliations:** 1 Kaoshiung Medical University, Department of Psychiatry Kaohiung Municipal Ta-Tung Hospital, Kaohsiung, Taiwan

## Introduction

It is common for psychiatric patients reporting somatic symptoms of headache. For these years, psychiatric treatment for headache in Taiwan is usually by medications. However, biofeedback training is also well addressed as a functional intervention for headache.

## Aims

The aim of this study is to look for the relations of headache and physical signs. It's also the idea that clinical psychologists might help the patients to control or to prevent the headache by relaxation training.

## Methods

This study invited the OPD psychiatric adult-patients without organic or other head injuries. The data of heartbeat rate, galvanic skin response (GSR) and respiratory were measured under three states of conditions. The first state was the baseline state which required patients to imagine the unimportant trifles of daily life. The second stage was the stress stage, after the 5 minutes of stress stage, the rehabilitation stage started with relaxation.

## Results

There were 100 patients participated, 69% of participants reported headache. The significant differences (p< .05) between headache group and non-headache group in the respiratory rate of the baseline condition and the rehabilitation state were shown. The headache group tended to show the higher rate of breathing in these two states.

## Conclusion

It is interesting to find out that the headache group tended to hold the quicker breathing rate than the non-headache group under the conditions of non-stress states. It was pointed that, for headache patients should take breathing slowly under the conditions of non-stress state.

No conflict of interest.

